# Silica-Supported Zinc(II)–Schiff-Base Catalysts for Lactide Ring-Opening Polymerization: Influence of Support Morphology and Ligand Substituents

**DOI:** 10.3390/polym18060737

**Published:** 2026-03-18

**Authors:** Darío M. González, Felipe Picero, Ornella Fuentes, Jocelyn Oyarce, Enrique Blázquez-Blázquez

**Affiliations:** 1 Instituto de Química, Pontificia Universidad Católica de Valparaíso, Campus Curauma, Valparaíso 2340000, Chile; 2 Instituto de Ciencia y Tecnología de Polímeros (ICTP-CSIC), Juan de La Cierva 3, 28006 Madrid, Spain

**Keywords:** ring-opening polymerization, lactide, zinc Schiff-base catalysts, silica-supported catalysts, poly(lactic acid)

## Abstract

Silica-supported zinc (II)–Schiff-base complexes were prepared through a simple and high-yield immobilization strategy and evaluated as heterogeneous catalysts for the ring-opening polymerization (ROP) of lactide. Silica gel and silica nanoparticles were employed as supports to assess the influence of support morphology and textural properties on catalytic performance. Comprehensive characterization by AAS, BET, SEM, and SEM–EDS confirmed effective anchoring of the Zn complexes, homogeneous metal distribution, and support-dependent textural modifications. The supported catalysts were active in the bulk ROP of racemic and enantiopure lactide, affording PLA with high conversions and moderate dispersities. Silica-gel-supported systems exhibited high and reproducible activity over a wide range of conditions, whereas catalysts supported on silica nanoparticles showed a stronger dependence on reaction time and ligand electronic effects, highlighting the key role of the support in modulating active site accessibility and chain growth. Microstructural and thermal analyses confirmed the formation of atactic PLA from rac-lactide and stereoregular PLLA from L-lactide. Overall, this study demonstrates that silica-supported zinc(II)–Schiff-base complexes constitute an effective and versatile heterogeneous platform for lactide ROP and underscore the importance of support properties in the rational design of sustainable catalysts for biodegradable polyester synthesis.

## 1. Introduction

The increasing environmental concerns associated with the excessive use of petroleum-derived plastics have stimulated great interest in the development of biodegradable polymers as sustainable alternatives [1,2,3]. Among them, poly(lactic acid) (PLA) has emerged as one of the most attractive candidates due to its biodegradability, biocompatibility, and mechanical properties that make it suitable for packaging, biomedical devices, and agricultural applications [4,5,6,7]. The large-scale production of PLA relies on the ring-opening polymerization (ROP) of lactide, which enables the synthesis of high-molecular-weight materials with well-controlled microstructures [8].

The catalytic system plays a crucial role in controlling the polymerization process and, consequently, the properties of the final polymer. Traditionally, tin(II) octoate (Sn(Oct)_2_) has been the most widely used industrial catalyst for lactide polymerization due to its high activity and ability to produce polymers with relatively narrow molecular weight distributions [9]. However, its toxicity and potential leaching into the final product raise significant health and environmental concerns, especially for biomedical and food-related applications [10,11]. In response, numerous homogeneous catalysts based on aluminum, titanium, and zinc complexes have been explored [12,13,14,15]. Recent studies have highlighted significant advances in Group IV complexes bearing tailored nitrogen-based ligands, as well as in aluminum systems supported by multidentate frameworks, which exhibit enhanced activity and improved control in lactide ROP [16,17,18,19]. These developments demonstrate the continuous progress in homogeneous catalytic design for cyclic ester polymerization. While these catalysts offer good activity and selectivity, their homogeneous nature complicates catalyst recovery and reuse, thus limiting their large-scale and environmentally friendly application.

To overcome these limitations, the development of heterogeneous catalysts for lactide ROP has received increasing attention. Beyond catalyst recovery considerations, immobilization of molecular complexes onto inorganic supports offers an opportunity to modulate their local coordination environment, dispersion, and accessibility under bulk polymerization conditions [20]. Among different supports, silica stands out due to its high surface area, chemical stability, and well-defined surface functionalities capable of interacting with metal centers [21,22,23]. Recent studies have demonstrated the successful ROP of lactide directly on silica surfaces, highlighting the relevance of support effects in determining catalytic behavior [21,22,24]. Thus, heterogenization can serve not only as a practical modification but also as a platform to explore structure–activity relationships in surface-confined catalytic systems.

In this context, Schiff-base complexes of zinc(II) represent promising candidates for the design of new catalytic systems. Schiff-base ligands are versatile, tunable, and capable of stabilizing zinc centers in active coordination environments [25,26]. Recent studies have reported highly active zinc complexes for the ROP of cyclic esters, demonstrating improved activity and control through tailored ligand design [27,28]. Additionally, several recent reviews have summarized their effectiveness in the ring-opening polymerization of cyclic esters, including lactide, emphasizing the influence of ligand design and metal coordination on activity and selectivity [25,26,29,30]. Zinc, in turn, is relatively non-toxic, abundant, and well-known for its catalytic activity in lactide polymerization [31]. Despite their potential, zinc(II)–Schiff-base complexes supported on silica have been scarcely investigated as heterogeneous catalysts for lactide ROP. Their exploration could provide a valuable alternative, combining efficient catalytic performance with easy recovery and recyclability.

To contextualize the performance of the catalytic systems investigated in this study, Table 1 summarizes representative examples of lactide ROP reported in the literature using selected metal-based catalysts under various conditions.

Direct comparison between catalytic systems is inherently limited due to differences in ligand structure, support properties, catalyst loading, and reaction conditions. The table is therefore intended to provide representative performance ranges rather than strict benchmarking. Notably, silica-supported systems reported in the literature typically afford molecular weights in the low-to-moderate kilodalton range under bulk conditions, placing the results of the present study within the range commonly reported for comparable heterogeneous systems.

Herein, we report the synthesis and characterization of heterogeneous catalysts based on zinc(II)–Schiff-base complexes immobilized on silica and their application in the ring-opening polymerization of lactide. The supported systems were prepared through a simple and high-yield impregnation methodology, enabling efficient anchoring of the catalytic species while preserving their activity. The influence of ligand substituents and support morphology on catalytic performance was systematically investigated, and the catalysts were evaluated in terms of relative activity, molecular weight control, dispersity, and resulting polymer microstructure and thermal properties under bulk polymerization conditions. Overall, the results demonstrate that silica-supported zinc(II)–Schiff-base complexes constitute a robust and sustainable platform for lactide ring-opening polymerization.

## 2. Materials and Methods

### 2.1. Materials

*rac*-Lactide (*rac*-LA) and L-lactide (L-LA) were purchased from Sigma-Aldrich (St. Louis, MO, USA) and purified by recrystallization from dry toluene, followed by drying under vacuum prior to use. Substituted aromatic aldehydes (2-hydroxybenzaldehyde, 2-hydroxy-4-methoxybenzaldehyde and 2-hydroxy-5-nitrobenzaldehyde), zinc acetate dihydrate (Zn(OAc)_2_·2H_2_O) and aminopropyltrimethoxysilane (APTMES) were obtained from Sigma-Aldrich and used as received. Toluene and methanol were supplied by Merck (Darmstadt, Germany) and used without further purification. Silica gel 60 (particle size 0.063–0.200 mm) was purchased from Merck, and silica nanoparticles (Aerosil^®^ 200, Degussa AG, Frankfurt am Main, Germany) were used as received.

### 2.2. Instrumentation and Characterization

^1^H NMR spectra were recorded at 298 K on a Bruker Avance Neo 400 MHz spectrometer (Billerica, MA, USA) and referenced to the residual solvent signal of CDCl_3_ (δ = 7.26 ppm). Molecular weights and dispersity values were determined by gel permeation chromatography (GPC) using a Shimadzu DGU-20A3R system (Kyoto, Japan) equipped with a Shimadzu RID-10A refractive index detector and a two-column set (Shim-pack GPC-800DP and GPC-80MD), calibrated with polystyrene standards. Prior to GPC and NMR analyses, the polymer solutions were filtered through 0.20 µm nylon syringe filters to remove any residual solid support. Differential scanning calorimetry (DSC) measurements were carried out on a TA Instruments Q100 calorimeter (New Castle, DE, USA) under a nitrogen atmosphere at a heating rate of 10 °C·min^−1^. Scanning electron microscopy (SEM) images and energy-dispersive X-ray spectroscopy (EDS) analyses were obtained using a HITACHI SU3500 scanning electron microscope (Tokyo, Japan) equipped with a Bruker X-Flash 410 M EDX detector (Billerica, MA, USA). Textural properties of the silica materials, including Brunauer–Emmett–Teller (BET) surface areas and pore volumes, were determined from N_2_ adsorption–desorption isotherms measured at −196 °C on a Micromeritics ASAP 2010 analyzer (Norcross, GA, USA). Zinc contents in the supported catalysts were quantified by atomic absorption spectroscopy (AAS) using a Shimadzu AA-6800 spectrophotometer (Kyoto, Japan) with an air–acetylene flame; samples were digested in concentrated HNO_3_, filtered and diluted to a known volume prior to analysis, and zinc concentrations were determined by external calibration according to the standard method SM 3111B [33] using reagent blanks.

### 2.3. Immobilization of Zn(ii)–Schiff-Base Catalysts on Silica

**Scheme 1:**
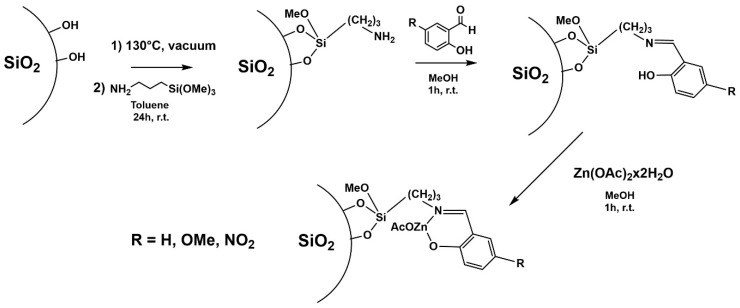
General procedure for the functionalization of silica and immobilization of Zn(II)–Schiff-base catalysts.

Silica gel and silica nanoparticles (Nps) were used as supports and treated following the same general procedure. The silica materials were first dehydrated under vacuum at 130 °C and then suspended in anhydrous toluene containing aminopropyltrimethoxysilane (APTMES) and stirred at room temperature for 24 h. The resulting amino-functionalized solids were recovered by filtration, washed with solvent, and dried under vacuum at 80 °C. In situ formation of the Schiff-base ligands (L) was achieved by contacting the amino-modified silica with methanolic solutions of the corresponding substituted aromatic aldehydes (2-hydroxybenzaldehyde, 2-hydroxy-4-methoxybenzaldehyde or 2-hydroxy-5-nitrobenzaldehyde) and stirring at room temperature for 1 h. Subsequently, Zn(II) was incorporated by treating the solid with a methanolic solution of zinc acetate dihydrate (Zn(OAc)_2_·2H_2_O), and the suspension was stirred at room temperature for 1 h. The resulting solids were filtered, washed with fresh solvent and dried to afford the silica-supported Zn(II)–Schiff-base catalysts. The procedure was applied analogously to both supports, yielding catalysts differing in the nature of the ligand substituent (H, OMe or NO_2_). The overall functionalization and immobilization procedure is summarized in Scheme 1.

### 2.4. Lactide Polymerization

Polymerization reactions were carried out in the melt state of the monomer. In a typical experiment, 1.5 g of rac-LA or L-LA (10.4 mmol) was placed in a round-bottom flask equipped with a magnetic stirring bar. The required amount of the supported catalyst was then added, calculated according to the desired monomer-to-zinc molar ratio ([LA]/[Zn]) based on the Zn content previously determined by AAS. The flask was purged with argon for several minutes and subsequently immersed in a thermostated oil bath at 130 °C, where the mixture was stirred for the selected polymerization time. After completion, the flask was removed from the oil bath, cooled to room temperature, and exposed to air. An aliquot of the reaction mixture was analyzed by ^1^H NMR spectroscopy to determine the monomer conversion. The [LA]/[Zn] ratios, reaction times, and catalyst systems used in the polymerization tests are summarized in Section 3.

## 3. Results and Discussion

### 3.1. Characterization of Supported Systems

**Figure 1:**
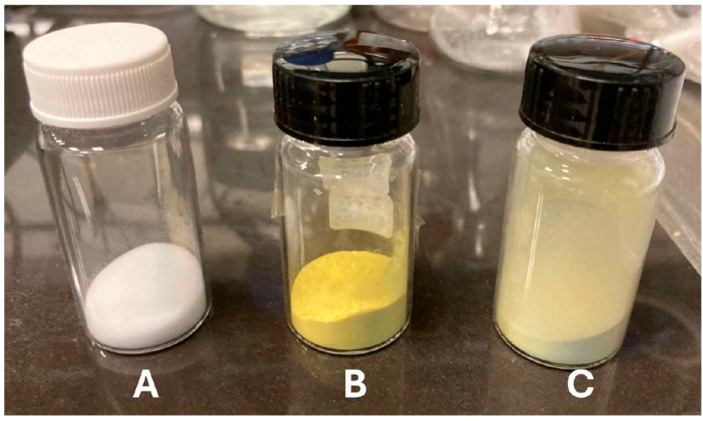
Example of silica gel at different modification stages: (**A**) dry silica gel; (**B**) silica gel functionalized with the ligand (Schiff base); and (**C**) supported catalytic system with a coordinated Zn atom.

As shown in Figure 1, a clear color change is observed upon silica functionalization, from the characteristic white of pristine silica to an intense yellow, which is associated with the formation of aromatic imine groups on the surface. After metal incorporation, the system exhibits a lighter coloration, a characteristic feature of coordination with a d^10^ metal such as Zn, which does not display electronic transitions responsible for color.

The supported catalytic systems were characterized by atomic absorption spectroscopy (AAS) after acid digestion of the samples in order to determine the incorporated zinc concentration. In addition, their textural properties, obtained from BET analysis, are summarized in Table 2, which includes both silica-gel-based materials and those supported on silica Nps. For the systems supported on silica gel, the zinc contents determined by AAS were very similar among the different functionalized samples, with values close to 3 wt%. When compared with unmodified silica gel, these materials exhibit a significant decrease in surface area as well as in pore volume and pore size. This behavior suggests that surface functionalization and subsequent anchoring of the metal complex occur predominantly within the porosity of the support. In this regard, silica-gel-based systems can be considered comparable in terms of metal loading and textural properties. Additionally, the Zn contents estimated by SEM–EDS are in good agreement with those determined by AAS, showing similar orders of magnitude and consistent trends among the different supported systems, despite the surface-sensitive and semi-quantitative nature of the EDS analysis.

On the other hand, catalytic systems supported on silica Nps exhibit a textural behavior clearly different from that of silica-gel-based materials. In these cases, the zinc contents determined by AAS fall within a slightly lower range, with values between 2.1 and 2.5 wt%. Compared to the unmodified Nps-SiO_2_ support, functionalization and subsequent metal incorporation lead to a marked decrease in surface area. However, unlike what is observed for silica gel, a significant increase in pore volume and pore size is recorded, a behavior that can be associated with surface modification of the support and partial agglomeration of the nanoparticles.

**Figure 2:**
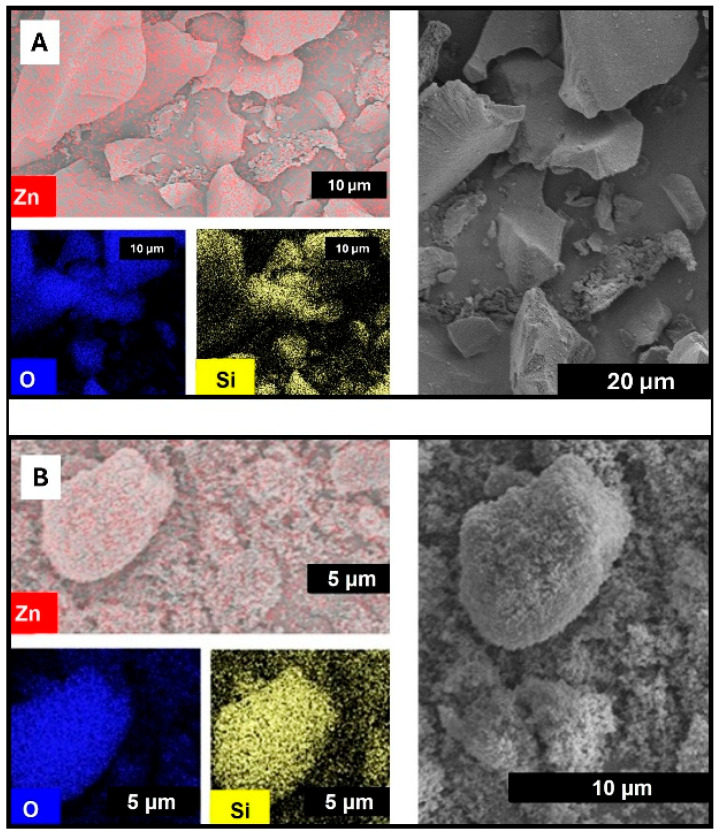
SEM micrographs and EDS elemental maps of Zn-functionalized supports: (**A**) silica gel and (**B**) silica Nps.

SEM micrographs reveal marked morphological differences between the studied supports, with silica gel (Figure 2A) displaying irregular micrometric particles without significant structural changes after functionalization, whereas silica Nps (Figure 2B) exhibit micrometric aggregates composed of primary nanoparticles, indicating a higher degree of agglomeration and a more complex hierarchical structure. These morphological features correlate well with the BET results, as the decrease in specific surface area and pore volume observed for functionalized silica gel can be mainly attributed to partial occupation of the intrinsic porosity of the support, consistent with the SEM observations, while in silica Nps, the surface modification induces more pronounced changes, compatible with the generation of interparticle porosity within the aggregates. SEM–EDS analysis confirms the presence of Zn in both supports, showing a homogeneous distribution of the metal on silica gel and silica Nps, with no evidence of segregation or Zn-rich domains, in agreement with the metal loading trends determined by AAS.

### 3.2. Reactivity Studies of Catalytic Systems in ROP of Lactide

In order to evaluate the reactivity of the Zn-based heterogeneous catalytic systems in the ring-opening polymerization (ROP) of lactide, catalytic tests were carried out using racemic lactide (rac-LA) and, additionally, L-lactide (L-LA), varying the monomer-to-catalyst ratio and the reaction time. The results are summarized in Table 3, which reports the conversions, molecular weights (Mn determined by GPC and NMR), and dispersity (Đ) obtained for the catalysts supported on silica gel, allowing the effect of the ligand substituent (H, OMe, and NO_2_) to be compared under equivalent conditions.

In the present systems, no external alcohol was added during polymerization. Considering that the supported complexes were prepared from Zn(OAc)_2_·xH_2_O (Scheme 1), the acetate ligand is likely retained in the coordination sphere of Zn. Based on precedents in the literature for Zn–acetate Schiff-base complexes in lactide ROP, particularly those reported by Jones et al. [34] a coordination–insertion mechanism is proposed. In this scenario, lactide first coordinates to the Zn center, followed by nucleophilic attack of a Zn–acetate species or a Zn–alkoxide formed in situ (e.g., via interaction with residual surface silanols or trace protic species), generating the active Zn–alkoxide propagating species. Subsequent monomer insertions proceed via the classical coordination–insertion pathway. The moderate Mn values and the deviation from ideal linear Mn–conversion relationships are consistent with non-simultaneous initiation and heterogeneous site distribution.

**Table 3:** Ring-opening polymerization (ROP) of lactide catalyzed by silica-gel-supported systems.

Entry	Catalyst	Monomer	[LA]:[Zn]	t (h)	Conv. ^a^ (%)	Mn ^b^ (kg/mol) (NMR)	Mn ^c^ (kg/mol) (GPC)	Đ ^c^ (GPC)
1	SiO_2_–Zn(L–OMe)	*rac*-LA	800	18	86	3.56	3.82	1.48
2	SiO_2_–Zn(L–NO_2_)	*rac*-LA	800	18	97	3.96	4.94	1.43
3	SiO_2_–Zn(L–H)	*rac*-LA	800	18	96	1.70	1.93	1.25
4	SiO_2_–Zn(L–OMe)	*rac*-LA	400	4	74	3.63	2.32	1.56
5	SiO_2_–Zn(L–NO_2_)	*rac*-LA	400	4	30	1.20	1.45	1.15
6	SiO_2_–Zn(L–H)	*rac*-LA	400	4	76	1.14	1.32	1.08
7	SiO_2_–Zn(L–OMe)	*rac*-LA	400	18	95	1.61	2.34	1.24
8	SiO_2_–Zn(L–NO_2_)	*rac*-LA	400	18	96	2.00	5.46	1.37
9	SiO_2_–Zn(L–H)	*rac*-LA	400	18	95	3.16	6.92	1.46
10	SiO_2_–Zn(L–OMe)	L-LA	400	18	96	2.24	2.97	1.35
11	SiO_2_–Zn(L–NO_2_)	L-LA	400	18	96	2.98	3.39	1.26

Reaction conditions: temperature = 130 °C, 1.5 g of *rac*-LA. ^a^ Conversion percentage determined by ^1^H NMR. ^b^ Average molecular weight determined by ^1^H NMR. ^c^ Average molecular weight and polydispersity index determined by GPC calibrated versus polystyrene standards and corrected by a factor of 0.58 according to recommendations from the literature [35,36].

To assess the effect of the support on catalytic performance, the results corresponding to the systems supported on silica Nps are presented in Table 4, under analogous experimental conditions to those employed for the silica-gel-based materials. This comparison allows the influence of support morphology and textural properties on both activity and polymerization control to be evaluated.

The results obtained for the catalysts supported on silica gel (Table 3) show that all systems are active in the ring-opening polymerization (ROP) of lactide, reaching high-to-near-quantitative conversions under the studied conditions. At a monomer-to-catalyst ratio of [LA]:[Zn] = 800 and a reaction time of 18 h, conversions above 85% are achieved for all three systems, indicating that immobilization of the Zn complex on silica gel does not significantly inhibit its catalytic activity. For comparison purposes, apparent turnover frequencies (TOF_app) were estimated based on total Zn loading and overall monomer conversion. Under representative conditions ([LA]:[Zn] = 800, 18 h), TOF_app values in the range of 38–43 h^−1^ were obtained. The effect of the ligand substituent is mainly reflected in the molecular weight values and, to a lesser extent, in the conversion, with the SiO_2_–Zn(L–NO_2_) and SiO_2_–Zn(L–OMe) systems tending to generate polymers with higher Mn values compared to SiO_2_–Zn(L–H). This trend suggests that the electronic nature of the ligand influences the propagation efficiency of the polymerization process, even in a heterogeneous environment. Upon decreasing the [LA]:[Zn] ratio to 400, a general increase in conversion with reaction time is observed, reaching near-quantitative values after 18 h. At shorter reaction times ([LA]:[Zn] = 400, 4 h), higher apparent TOF values (30–76 h^−1^) were calculated, consistent with faster initial propagation rates and comparable to values reported for heterogeneous bulk ROP systems [22,29]. Nevertheless, the experimental Mn values do not always follow a linear relationship with conversion, which may indicate non-simultaneous initiation of the active sites or the occurrence of secondary processes, such as transesterification, phenomena commonly reported for heterogeneous systems [5,35]. consistent with the moderate Mn values typically observed in heterogeneous bulk ROP systems. At prolonged reaction times (18 h), the catalysts supported on silica gel reach high conversions regardless of the ligand substituent, whereas at shorter reaction times, more pronounced differences in activity are observed, indicating that the electronic effect of the ligand mainly manifests during the initial stages of the process. The Mn values and dispersities obtained fall within the range expected [24,29] for a heterogeneous ROP and show good agreement between the values determined by NMR and GPC, supporting the consistency of the experimental data.

Finally, regarding dispersity, the values remain within a moderate range (Đ ≈ 1.1–1.5), which is consistent with a heterogeneously catalyzed ROP process and suggests a reasonable degree of control over chain growth. Likewise, the results obtained using L-lactide (L-LA) are comparable to those observed with rac-LA, indicating that the catalysts supported on silica gel exhibit robust activity toward both types of monomer. In addition, the use of L-LA enables access to potentially stereoregular polymers, whose microstructure and properties will be analyzed in subsequent sections.

On the other hand, the results obtained for the catalysts supported on silica Nps (Table 4) evidence a catalytic behavior distinct from that observed for silica-gel-based systems. In general, these materials exhibit more scattered conversions and a stronger dependence on both reaction time and the nature of the ligand substituent, highlighting a more pronounced influence of the support on catalytic performance.

At a [LA]:[Zn] ratio of 800, the Nps–SiO_2_–Zn(L–H) and Nps–SiO_2_–Zn(L–OMe) systems show moderate activity, particularly at short reaction times, reaching limited conversions and relatively low Mn values. In contrast, the Nps–SiO_2_–Zn(L–NO_2_) system exhibits significantly higher activity, achieving near-quantitative conversions after 18 h and producing polymers with Mn values comparable to those obtained with silica-gel-supported catalysts. Under these conditions ([LA]:[Zn] = 800, 18 h), apparent TOF values in the range of 22–43 h^−1^ were calculated, depending on the ligand substituent, whereas at shorter reaction times ([LA]:[Zn] = 800, 4 h) higher apparent TOF values (58–106 h^−1^) were observed, reflecting the stronger time dependence of these systems. This behavior suggests that the electronic character of the ligand plays a key role in monomer activation and chain propagation within the silica Nps environment. Consistently, catalysts supported on silica Nps tend to generate polymers with systematically lower Mn values than their silica-gel-supported counterparts, even when high conversions are reached. This effect is more pronounced at short reaction times and can be associated with the morphological and textural features of the support, which modulate the effective accessibility of the active sites and chain growth, in agreement with the BET and SEM results. Despite these differences, the dispersities obtained for silica-Np-based systems remain within a moderate range (Đ ≈ 1.2–1.4), indicating that the ROP process retains a reasonable degree of control. Overall, these results confirm that silica Nps enable effective immobilization of the catalytic systems, although the support exerts a significant influence on the accessibility and efficiency of the active sites, thereby modulating chain growth and overall catalytic performance.

Overall, the polymerization results highlight that the nature of the support plays a decisive role in the catalytic performance of Zn-based systems. While catalysts supported on silica gel exhibit reproducible and efficient activity under bulk conditions, with more efficient chain growth over a wide range of conditions, silica-Np-based systems show a greater sensitivity to both reaction time and the electronic character of the ligand. These differences can be attributed to the morphological and textural properties of the supports, which modulate the accessibility of the active sites and monomer diffusion, as evidenced by BET and SEM analyses. The apparent TOF values estimated for representative conditions further support the influence of support morphology on catalytic efficiency. Nevertheless, both supports enable effective immobilization of the catalytic complexes and lead to polymers with moderate dispersities, confirming the viability of these heterogeneous systems for the ROP of lactide. For comparison purposes, polymerization experiments using the corresponding unsupported molecular Zn–salen (N_2_O_2_) complexes tested under bulk (melt) conditions, which are soluble under these reaction conditions, were also performed under identical parameters and are provided in the Supporting Information (Table S1). This comparison serves as an internal activity benchmark under equivalent homogeneous melt conditions rather than as a direct structural analogy with the supported half-salen (N,O) systems described herein. Additionally, blank polymerization experiments using bare silica gel and silica nanoparticles were performed under identical conditions to evaluate whether the support itself could promote lactide polymerization. The resulting ^1^H NMR spectra, which are dominated by signals of unreacted lactide, are provided in the Supporting Information (Section S3.4).

### 3.3. Polymer Microstructure and Thermal Properties

**Figure 3:**
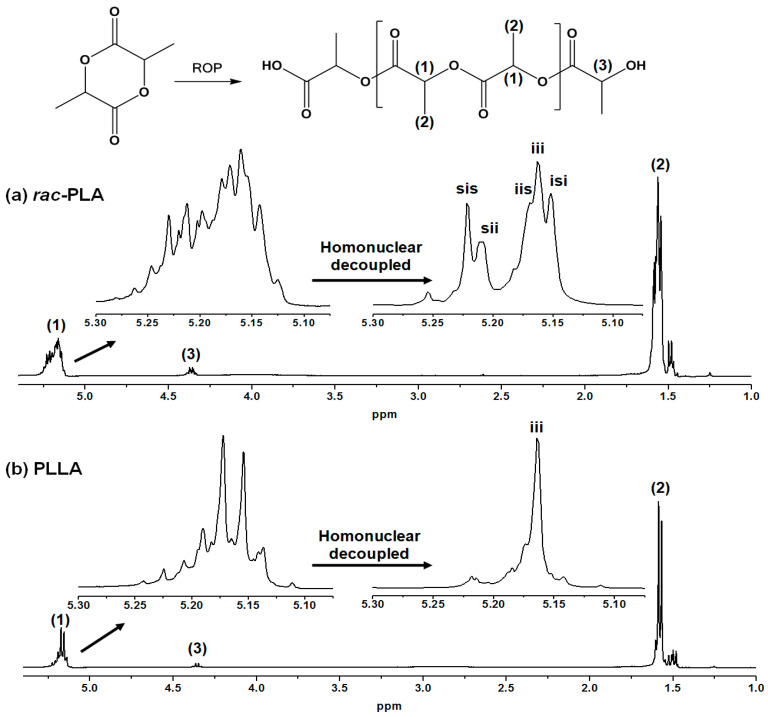
^1^H NMR spectra of *rac*-PLA and PLLA in the methine region before and after homonuclear decoupling, illustrating the different tacticity sequences.

In order to evaluate the microstructure of the polymers obtained by ROP of lactide, ^1^H NMR analysis with homonuclear decoupling was performed. This technique allows for the resolution of signals associated with the different tacticity sequences along the PLA backbone, enabling the identification and quantification of stereochemical distributions. Figure 3 shows representative spectra of *rac*-PLA and PLLA, highlighting the differences in the multiplicity of the methine proton signals, which are characteristic of a statistical microstructure in *rac*-PLA and a stereoregular microstructure in the polymer derived from L-lactide. It should be explicitly noted that the signal labeled (3) at δ ≈ 4.3–4.4 ppm corresponds to the terminal methine proton of PLA adjacent to the carboxylic acid end group and does not arise from residual open-chain lactide.

**Figure 4:**
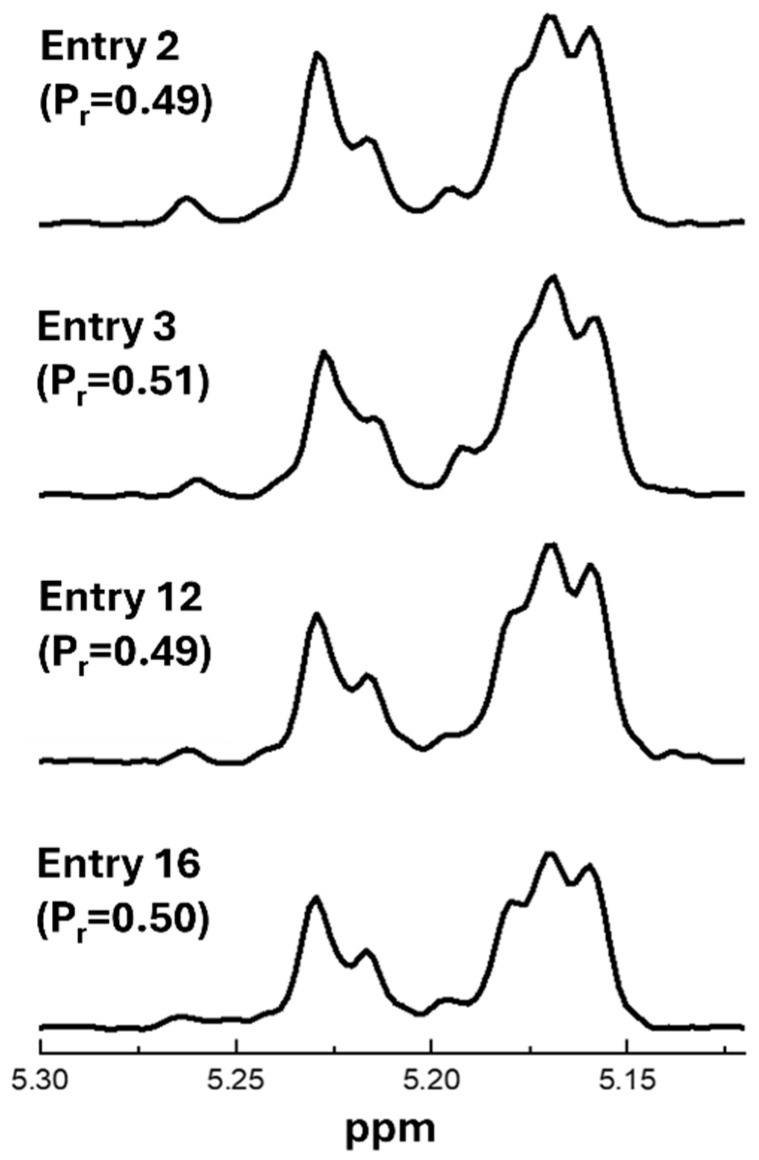
^1^H NMR spectra (methine region) of representative *rac*-PLA samples obtained using silica-gel- and silica-Np-supported catalysts (Entries 2, 3, 12 and 16) after homonuclear decoupling.

Representative *rac*-PLA samples were selected under identical reaction conditions ([LA]:[Zn] = 800, 18 h) for both silica-gel- and silica-Np-supported catalysts in order to assess the influence of the support and ligand on polymer microstructure. As shown in Figure 4, all representative *rac*-PLA samples exhibit very similar methine-region ^1^H NMR patterns after homonuclear decoupling, characterized by comparable distributions of tacticity sequences. The calculated Pr values are close to 0.5 for all samples, indicating a statistical microstructure and the absence of stereocontrol, regardless of the nature of the ligand or the support employed.

**Figure 5:**
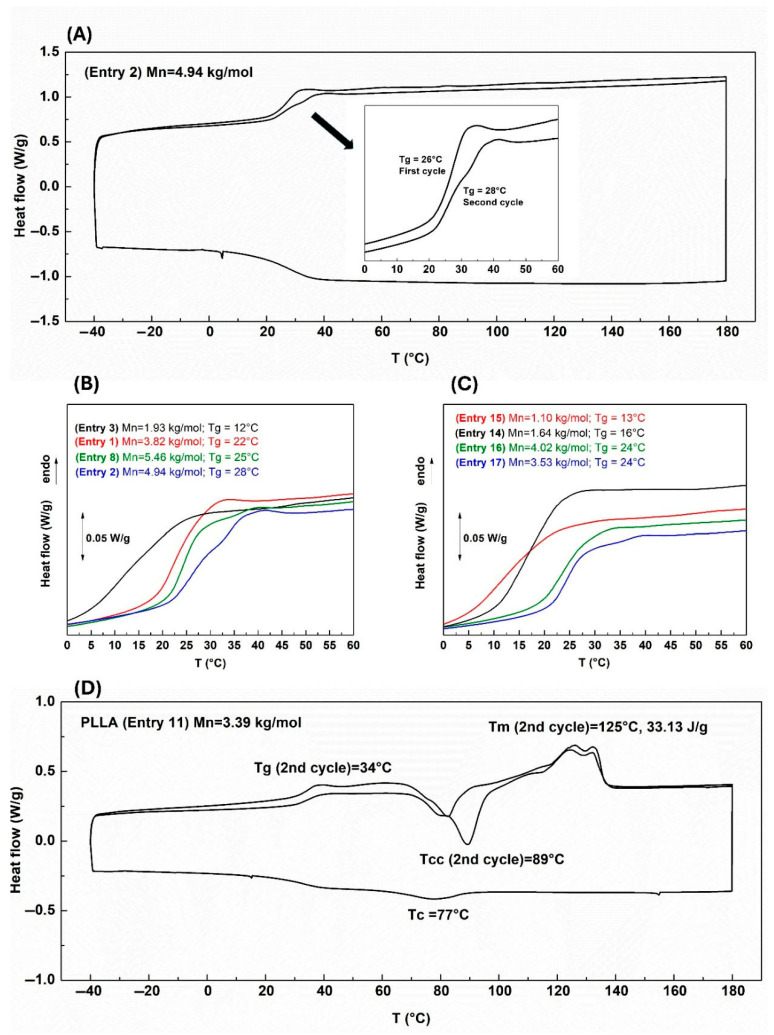
DSC thermograms of representative PLA samples. (**A**) Heating–cooling–second heating cycle of *rac*-PLA (Entry 2). (**B**) Enlarged Tg region (second heating) for *rac*-PLA obtained with silica-gel-supported catalysts. (**C**) Enlarged Tg region (second heating) for *rac*-PLA obtained with silica-Np-supported catalysts. (**D**) DSC thermogram of PLLA (Entry 11).

To further correlate the microstructural features of the obtained polymers with their thermal behavior, DSC analysis was carried out on representative PLA samples. Figure 5 summarizes the thermal response of *rac*-PLA and PLLA obtained using the supported Zn catalysts, highlighting the influence of molecular weight and polymer microstructure on the observed thermal transitions.

For rac-PLA samples, DSC thermograms display a single glass transition, with no evidence of crystallization or melting events over the explored temperature range (Figure 5, panels A–C). This behavior is consistent with the statistical microstructure inferred from ^1^H NMR analysis (Pr ≈ 0.5), which hinders chain packing and crystallization. A slight increase in Tg is observed from the first to the second heating cycle (Figure 5A), attributable to the removal of thermal history and physical relaxation effects. Under representative polymerization conditions ([LA]/[Zn] = 400), the amount of heterogeneous catalyst employed corresponds to approximately 3 wt% inorganic residue relative to the polymer mass. Such a relatively low loading is below the range typically required to induce significant nucleating effects in PLA-based systems and is therefore not expected to substantially influence the crystallization behavior of rac-PLA, which is predominantly governed by its low stereoregularity and moderate molecular weight.

As illustrated in Figure 5B,C, Tg values determined from the second heating cycle increase with increasing Mn for both silica-gel- and silica-Np-supported systems, indicating that molecular weight is the dominant factor governing the glass transition temperature in these materials, irrespective of the nature of the support. In contrast, PLLA exhibits well-defined glass transition, crystallization and melting transitions (Figure 5D), reflecting its stereoregular microstructure and confirming the strong relationship between polymer microstructure and thermal properties. In this case, the thermal behavior is primarily dictated by the intrinsic stereoregularity of PLLA and its molecular weight rather than by the small inorganic fraction present.

## 4. Conclusions

Silica-supported zinc(II)–Schiff-base complexes were successfully prepared through a simple and high-yield immobilization strategy, affording robust heterogeneous catalysts for the ring-opening polymerization (ROP) of lactide. Structural and textural analyses confirmed effective anchoring of the Zn complexes on both silica gel and silica nanoparticle supports, with homogeneous metal distribution and support-dependent textural features. The nature of the support was found to play a decisive role in catalytic performance, as silica-gel-supported systems exhibited high and reproducible activity under bulk conditions, affording polymers with moderate molecular weights and dispersities typical of heterogeneous ROP systems, whereas silica-nanoparticle-supported catalysts showed a stronger dependence on reaction time and ligand electronic effects. The electronic character of the Schiff-base ligands influenced molecular weight development and propagation efficiency even under heterogeneous conditions, while all systems enabled controlled lactide polymerization with moderate dispersities. Microstructural and thermal analyses confirmed the formation of atactic PLA from rac-lactide and stereoregular PLLA from L-lactide. Overall, these results demonstrate that silica-supported zinc(II)–Schiff-base complexes constitute an effective and versatile heterogeneous platform for lactide ring-opening polymerization and highlight the importance of support properties in the rational design of sustainable catalysts for biodegradable polyesters.

## Figures and Tables

**Table 1:** Representative examples of lactide ROP using selected metal-based catalytic systems.

Catalyst System	Nature	Conditions (T, Medium)	[LA]/[M]	Mn (kg·mol^−1^)	Đ	Ref.
Sn(Oct)_2_	Homogeneous	140 °C, bulk	13.5	1.30 *	-	[9]
Zn β-diiminate (ZnOiPr)	Homogeneous	20 °C, CH_2_Cl_2_	100	16.8	1.19	[11]
Zn β-diiminate on mesoporous silica	Supported	80 °C, toluene	58	1.29	1.12	[23]
Zn β-diiminate on silanol-capped silica	Supported	80 °C, toluene	132	3.25	1.42	[23]
Al–SiO_2_	Supported	130 °C, bulk	300	3.00	1.13	[32]
Zn–Schiff base/SiO_2_ (This work)	Supported	130 °C, bulk	400–800	1–7	1.1–1.5	This work

* For ref. [9], Mn was estimated from the reported DPn (^1^H NMR) using Mn ≈ DPn × 72.06 g·mol^−1^.

**Table 2:** Zinc loading and textural properties of the supported catalytic systems.

Support	Catalyst	[Zn] AAS (%*w*/*w*)	[Zn] SEM-EDS (%*w*/*w*)	Surface Area (m^2^/g)	Pore Volume (cm^3^/g)	Pore Size (nm)
Silica gel	SiO_2_	-	-	458	0.69	6.04
	SiO_2_–Zn(L–H)	3.28	3.60	302	0.41	5.39
	SiO_2_–Zn(L–OMe)	3.01	3.00	314	0.44	5.56
	SiO_2_–Zn(L–NO_2_)	3.05	3.10	281	0.40	5.70
Silica-Nps	Nps-SiO_2_	-	-	334	1.05	11.58
	Nps-SiO_2_–Zn(L–H)	2.14	2.04	152	1.53	35.64
	Nps-SiO_2_–Zn(L–OMe)	2.35	2.00	157	1.47	33.44
	Nps-SiO_2_–Zn(L–NO_2_)	2.50	2.37	151	1.16	27.76

**Table 4:** Ring-opening polymerization (ROP) of lactide catalyzed by silica-nanoparticle-supported systems.

Entry	Catalyst	Monomer	[LA]:[Zn]	t (h)	Conv. ^a^ (%)	Mn ^b^ (kg/mol) (NMR)	Mn ^c^ (kg/mol) (GPC)	Đ ^c^ (GPC)
12	Nps–SiO_2_–Zn(L–H)	*rac*-LA	800	18	87	0.50	0.63	1.27
13	Nps–SiO_2_–Zn(L–H)	*rac*-LA	800	4	29	0.32	0.42	1.24
14	Nps–SiO_2_–Zn(L–OMe)	*rac*-LA	800	18	49	1.20	1.64	1.35
15	Nps–SiO_2_–Zn(L–OMe)	*rac*-LA	800	4	45	0.80	1.10	1.31
16	Nps–SiO_2_–Zn(L–NO_2_)	*rac*-LA	800	18	96	3.10	4.02	1.29
17	Nps–SiO_2_–Zn(L–NO_2_)	*rac*-LA	800	4	53	1.90	2.41	1.24
18	Nps–SiO_2_–Zn(L–NO_2_)	*rac*-LA	400	18	96	2.80	3.53	1.28

Reaction conditions: temperature = 130 °C, 1.5 g of *rac*-LA. ^a^ Conversion percentage determined by ^1^H NMR. ^b^ Average molecular weight determined by ^1^H NMR. ^c^ Average molecular weight and polydispersity index determined by GPC calibrated versus polystyrene standards and corrected by a factor of 0.58 according to recommendations from the literature [35,36].

## Data Availability

The original contributions presented in this study are included in the article and Supplementary Materials. Further inquiries can be directed to the corresponding author.

## Supplementary Materials

The following supporting information can be downloaded at: https://www.mdpi.com/article/10.3390/polym18060737/s1. Figure S1. Synthetic scheme of Zn complexes. Table S1. Bulk ring-opening polymerization of lactide using unsupported molecular Zn–salen complexes tested under bulk (melt) conditions. Figure S2. Representative ^1^H NMR spectra of rac-PLA and PLLA samples containing residual lactide, highlighting the signals used for conversion determination. Figure S3. ^1^H NMR spectrum highlighting the terminal methine signal (3) adjacent to the carboxylic acid end group and the backbone methine signal (1) used for Mn determination by end-group analysis. Figure S4. ^1^H NMR spectrum of purified rac-lactide prior to polymerization (CDCl_3_). Figure S5. Comparison of the ^1^H NMR (CDCl_3_) spectra of control experiments and representative polymerization mixtures: (A) Blank experiment with silica gel; (B) Blank experiment with silica nanoparticles; (C) *rac*-PLA obtained at 95% conversion; (D) *rac*-PLA obtained at 74 % conversion.
