# Supplementary material for: Silica-Supported Zinc(II)–Schiff-Base Catalysts for Lactide Ring-Opening Polymerization: Influence of Support Morphology and Ligand Substituents
Source: Polymers (Basel). 2026 Mar 18;18(6):737. doi: 10.3390/polym18060737 (PMC13030640; doi:10.3390/polym18060737)
Supplement: Supplementary file 1 [file polymers-18-00737-s001.zip › polymers-4187311-supplementary.pdf]

Supporting Information

Silica-Supported Zinc(II) Schiff-Base Catalysts for Lactide Ring-Opening Polymerization: Influence of Support Morphology and Ligand Substituents

Darío M. González[1], Felipe Picero[1], Ornella Fuentes[1], Jocelyn Oyarce[1], Enrique Blázquez[2]

[1]Instituto de Química, Pontificia Universidad Católica de Valparaíso, Campus Curauma, Valparaíso, Chile.

[2]Instituto de Ciencia y Tecnología de Polímeros (ICTP-CSIC), Juan de la Cierva, 3 28006, Madrid, Spain.

dario.gonzalez@pucv.cl

This Supporting Information contains additional data related to the synthesis of homogeneous Zn complexes and their catalytic performance in the ROP of lactide, as well as representative NMR calculations.

S1. Synthesis of Homogeneous Zn Complexes

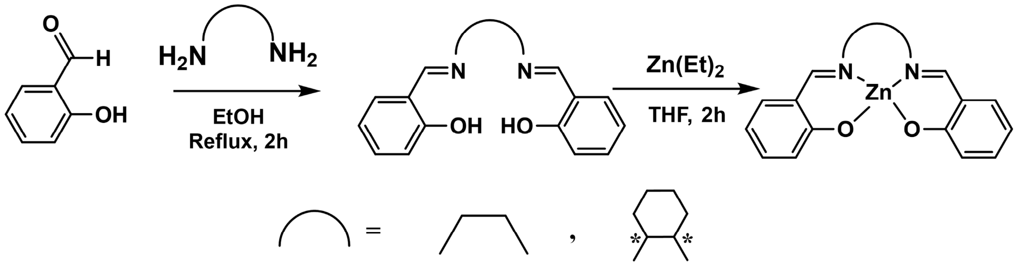

Figure S1. Synthetic scheme of Zn complexes.

The salen-type Schiff bases were synthesized by condensation of the corresponding diamine with the aromatic aldehyde in ethanol under reflux for 2 h, according to previously reported procedures. The ligands were obtained as yellow solids in yields above 90%.

The zinc complexes were prepared by dropwise addition of a solution of $ZnEt_2$ to a stirred solution of the ligand in THF at 0 °C. An immediate yellow precipitate formed upon addition, corresponding to the Zn(II) complex, which was found to be practically insoluble in common organic solvents at room temperature. However, under the bulk polymerization conditions employed in this work (molten lactide at 130 °C), the complexes dissolve in the monomer, providing a homogeneous reaction medium. The resulting solid was isolated by filtration, washed with cold THF, and dried under vacuum. The complexes were obtained in 91% yield for the cyclic diamine system and 74% yield for the ethylene-bridged system.

Due to their very low solubility, NMR characterization of the complexes was not feasible. The formation of the metal complexes was confirmed by electron ionization mass spectrometry (EI-MS). The complex $C_{16}H_{14}N_2O_2Zn$ exhibited an intense molecular ion peak at m/z = 330.03 (M^{+}•), consistent with the calculated mass (330.03 Da). The complex $C_{20}H_{20}N_2O_2Zn$ displayed a molecular ion at m/z = 384.08 (M^{+}•). In both cases, the characteristic isotopic pattern of zinc was clearly observed in the molecular ion region, together with minor fragments arising from partial fragmentation of the salen framework, supporting the proposed structures.

S2. Bulk Ring-Opening Polymerization

The results of the bulk ROP experiments performed using the homogeneous Zn–salen complexes are summarized in Table S1. The same parameters reported in the main manuscript (monomer-to-catalyst ratio, temperature, reaction time, conversion, Mn, and dispersity) are included to allow direct comparison with the supported catalytic systems.

Table S1. Bulk ring-opening polymerization of lactide using unsupported molecular Zn–salen complexes tested under bulk (melt) conditions.

Entry	Catalyst	Monomer	[LA]/[Zn]	t (h)	Conversion (%)	Mn (kg·mol^{-1})	Đ
1	Ethyl	*rac*-LA	400	4	60	2.02	1.11
2	Cyclohexyl	rac-LA	400	4	67	3.25	1.18
3	Ethyl	*rac*-LA	800	18	92	5.19	1.43
4	Cyclohexyl	*rac*-LA	800	18	86	3.18	1.20
5	Ethyl	L-LA	800	18	96	2.52	1.25
6	Cyclohexyl	L-LA	800	18	85	3.52	1.20

Polymerizations were performed under bulk conditions as described in the Experimental Section of the main manuscript. Conversion was determined by ^{1}H NMR spectroscopy. Mn and Đ were measured by GPC using polystyrene standards.

S3. NMR Determination of Conversion and Mn

S3.1. Monomer Conversion

Monomer conversion was determined by ^{1}H NMR spectroscopy by integrating the methine proton signal of unreacted lactide (δ ≈ 5.00 ppm) and comparing it with the methine proton signal of the PLA backbone (δ ≈ 5.15–5.20 ppm), as illustrated in Figure S2. The clear separation of these resonances allows independent integration of monomer and polymer signals in the reaction mixture.

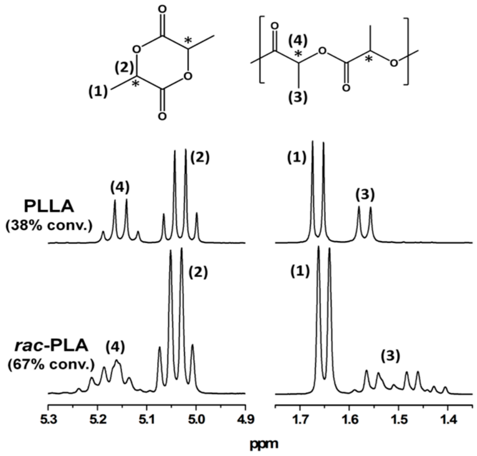

Figure S2. Representative ^{1}H NMR spectra of rac-PLA and PLLA samples containing residual lactide, highlighting the signals used for conversion determination.

The percentage of conversion was calculated according to Equation (S1):

$$\textbf{Conversion (\%)} = [1 - (I_{monomer} / (I_{monomer} + I_{polymer}))] \times 100$$

where $I_{monomer}$ and $I_{polymer}$ correspond to the integrated areas of the lactide and PLA methine signals, respectively.

S3.2. Mn Determination by End-Group Analysis

The number-average molecular weight (Mn,NMR) was determined by end-group analysis using the integral of the terminal methine proton adjacent to the carboxylic acid end group (signal (3), appearing as a quartet at δ ≈ 4.3–4.4 ppm) relative to the methine proton of the PLA repeating units (signal (1), δ ≈ 5.15–5.20 ppm), as shown in Figure S3.

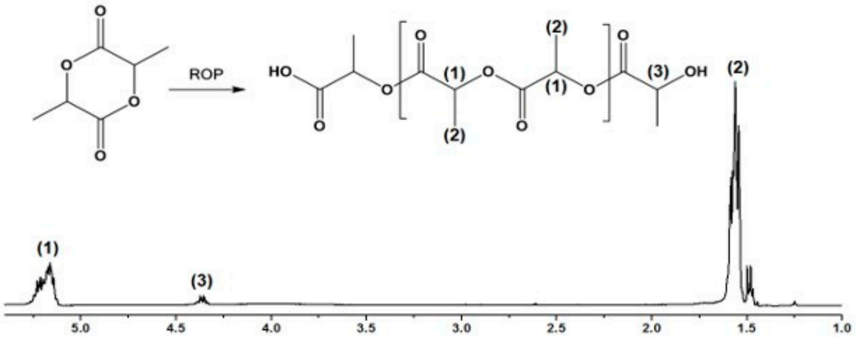

Figure S3. ^{1}H NMR spectrum highlighting the terminal methine signal (3) adjacent to the carboxylic acid end group and the backbone methine signal (1) used for Mn determination by end-group analysis.

Since each repeating unit contributes one methine proton and each polymer chain contains one terminal methine proton at the carboxylic acid end, the degree of polymerization (DP_n) was calculated as:

$$\mathbf{DP_n = I_{(1)} / I_{(3)}}$$

The number-average molecular weight was then obtained from:

$$\mathbf{Mn\ (NMR) = DP_n \times 72.06 + M_{end\ groups}}$$

where 72.06 g·mol^{-1} corresponds to the molar mass of the lactyl repeating unit.

S3.3 ^{1}H NMR characterization of *rac*-lactide monomer

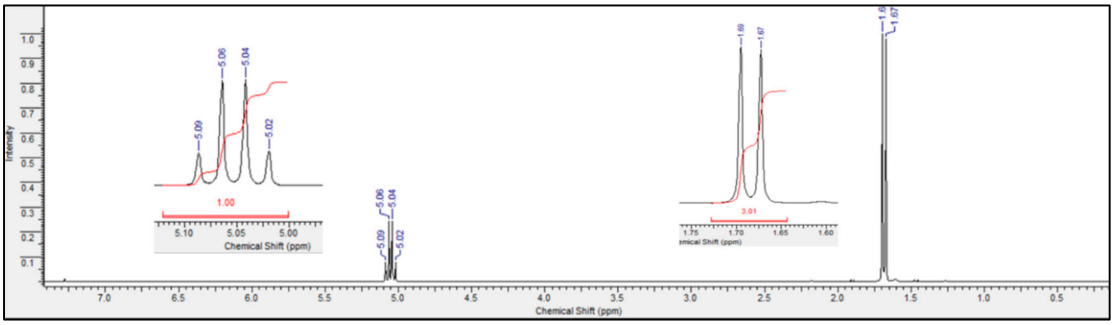

Figure S4. ^{1}H NMR spectrum of purified rac-lactide prior to polymerization ($CDCl_3$).

The absence of signals in the 4.2–4.4 ppm region confirms the absence of detectable open-chain species.

S3.4 Blank Polymerization Experiments Using Bare Silica Supports

Blank experiments using bare silica gel and silica nanoparticles were performed under the same reaction conditions (1.5 g *rac*-lactide, 130 °C, 1 h). Since no metal complex was present, the monomer-to-metal ratio could not be defined; therefore, the amount of silica support was fixed at 3 wt%, corresponding to the catalyst loading typically used for the supported systems. Figure S5 compares the resulting ^{1}H NMR spectra with those obtained from representative *rac*-lactide polymerization experiments.

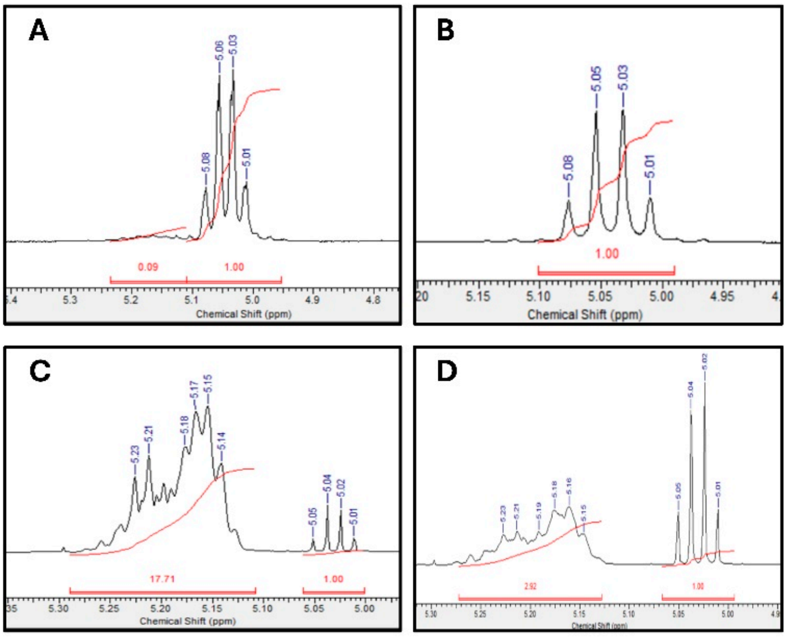

Figure S5. Comparison of the ^{1}H NMR ($CDCl_3$) spectra of control experiments and representative polymerization mixtures: (A) Blank experiment with silica gel; (B) Blank experiment with silica nanoparticles; (C) *rac*-PLA obtained at 95% conversion; (D) *rac*-PLA obtained at 74 % conversion.

The spectra of the blank experiments are dominated by the characteristic signals of unreacted lactide (methine ~5.05 ppm, methyl ~1.67 ppm), whereas the polymerization mixtures clearly display additional signals corresponding to PLA (methine ~5.15–5.20 ppm and methyl ~1.55–1.60 ppm). These results indicate that the silica supports alone do not promote significant lactide polymerization under the employed conditions.
